# Risk factors for endothelial cell loss after Descemet membrane endothelial keratoplasty (DMEK)

**DOI:** 10.1038/s41598-020-68023-0

**Published:** 2020-07-06

**Authors:** Takahiko Hayashi, Silvia Schrittenlocher, Sebastian Siebelmann, Viet Nhat Hung Le, Mario Matthaei, Jeremy Franklin, Björn Bachmann, Claus Cursiefen

**Affiliations:** 1 Department of Ophthalmology, University of Cologne, Cologne, Germany; 2 Department of Ophthalmology, Yokohama Minami Kyosai Hospital, Kanagawa, Japan; 3 Department of Ophthalmology, Hue College of Medicine and Pharmacy, Hue University, Hue, Vietnam; 4 Institute of Medical Statistics and Computational Biology, University of Cologne, Cologne, Germany; 5 Centre for Molecular Medicine Cologne, CMMC, University of Cologne, Cologne, Germany

**Keywords:** Diseases, Eye diseases, Corneal diseases

## Abstract

This study aimed to identify the risk factors for endothelial cell density (ECD) loss after Descemet membrane endothelial keratoplasty (DMEK) and analyse whether donor tissues from cold versus organ culture differ in terms of ECD loss after DMEK. Consecutive DMEK cases from a prospective database for Fuchs’ endothelial corneal dystrophy were retrospectively analysed between 2011 and 2016 at the University of Cologne, and the possible risk factors for ECD loss, including patient-related factors, type of tamponade (air or 20% sulphur hexafluoride gas), type of surgery (triple DMEK or DMEK alone), re-bubbling, immune rejection, and donor-related factors were determined. Eight hundred and forty-one eyes were selected. There was no significant difference in the best-corrected visual acuity (logarithm of the minimal angle of resolution) and corneal thickness (P = 0.540 and P = 0.667) between groups. Immune reactions were more common in cold cultures (P = 0.019), but ECD loss (1 year after DMEK) was greater in organ cultures (38.3 ± 0.8%) than in cold cultures (34.7 ± 1.4%) (P = 0.022). Only re-bubbling was significantly associated with ECD loss (P < 0.001). Re-bubbling was found to be a key factor for ECD loss at 1 year after DMEK.

## Introduction

Descemet membrane endothelial keratoplasty (DMEK) is a widely applied technique for performing keratoplasty and restoring vision in patients with endothelial diseases, such as Fuchs’ endothelial corneal dystrophy (FECD) or bullous keratopathy^1–5^. As this technique replaces only the endothelial layer that is attached to the Descemet membrane (DM), surgeons can achieve rapid improvements in visual acuity, better final visual outcomes, and less immunological reactions, compared to other techniques^6–9^, such as Descemet stripping automated endothelial keratoplasty (DSAEK) or penetrating keratoplasty (PKP).

Despite the excellent outcomes, there are a steep short-term decrease in the transplanted endothelial cell density (ECD) for up to 6 months and a gradual decrease after 6 months. The average ECD loss rate at 6 months described in the literature ranges from 29 to 48%^10–12^. Although many researchers have indicated certain factors associated with ECD loss in both DMEK and PKP or in DSAEK, such as graft diameter^13,14^, surgical indication, type of filling gas^15^, rebubbling, or culture medium^16–18^, there has been no consensus on the most important factor associated with the final ECD outcome.

Regarding the relationship between the condition of the culture and the surgical outcomes, it has been reported in a small number of short-term case series that cold cultures may negatively impact the outcomes^18^. Evaluations in larger cohorts are needed. Furthermore, the difference between organ culture and cold culture needs to be investigated.

This study aimed to identify the risk factors for endothelial cell loss after DMEK by evaluating the correlation between surgical outcomes and clinically relevant factors. To the best of our knowledge, this is the first study to evaluate the factors associated with ECD loss at 1 year after DMEK in a large sample.

## Results

### Patient demographics

Table 1 summarises the characteristics of the patients who underwent DMEK in the current study. A total of 841 eyes from 841 patients were included in this study, which consisted of 354 men and 487 women. The recipients’ mean age was 70.2 ± 9.1 years. All patients were diagnosed with FECD. A total of 464 (55.2%) patients received triple DMEK, whereas 377 (44.8%) patients were treated with DMEK alone. Regarding tamponade gas, air was used in 432 (51.4%) eyes and 20% sulphur hexafluoride (SF6) gas in 409 (48.6%) eyes.

**Table 1:** Characteristics of the patients.

Factors	Total	Organ culture	Cold culture	P-value (organ vs cold)
Numbers of eyes	841	644	197	
Sex: female (%)/male (%)	487 (57.9)/354 (42.1)	368 (57.1)/276 (42.8)	119 (60.4)/78 (39.6)	0.440
Age (years) (mean ± SD)	70.2 ± 9.1	69.8 ± 0.4	71.8 ± 0.6	0.008
Donor sex: female (%)/male (%)	286 (38.8)/451 (60.2)	212 (37.4)/355 (62.6)	74 (43.5)/96 (56.5)	0.150
Donor age (years) (mean ± SD)	69.0 ± 10.9	70.0 ± 0.4	65.9 ± 0.8	< 0.001
Storage time (days) (mean ± SD)	16.9 ± 6.1	18.7 ± 0.2	11.4 ± 0.4	< 0.001
Lens status: PC-IOL (%)	367 (43.9)	275 (43.2)	91 (46.7)	0.574
Type of surgery: triple DMEK (%)	464 (55.2)	358 (55.6)	106 (53.8)	0.692
Type of tamponade gas: SF6 (%)	408 (48.6)	298 (46.3)	110 (55.8)	0.028

*SD* standard deviation, *PC-IOL* posterior chamber intra-ocular lens, *DMEK* Descemet membrane endothelial keratoplasty, *triple DMEK* DMEK combined with cataract surgery and intra-ocular lens implantation, *SF6* sulphur hexafluoride.

### Clinical course

The best-corrected visual acuity (BCVA) (logarithm of the minimal angle of resolution [logMAR]) improved from 1.15 ± 0.72 logMAR (20/200 Snellen) preoperatively to 0.31 ± 0.27 logMAR (20/40 Snellen) at 1 month, 0.19 ± 0.17 logMAR (20/32 Snellen) at 3 months, 0.14 ± 0.10 logMAR (20/30 Snellen) at 6 months, and 0.11 ± 0.11 logMAR (20/25 Snellen) at 12 months postoperatively. The central corneal thickness (CCT) decreased from 664.5 ± 122.9 μm preoperatively to 564.3 ± 107.0 μm at 1 month, 533.1 ± 56.7 μm at 3 months, 533.4 ± 65.1 μm at 6 months, and 534.7 ± 45.2 μm at 12 months postoperatively. The donor corneal ECD decreased from 2,717 ± 218 cells/mm^2^ preoperatively to 1802 ± 343 cells/mm^2^ at 1 month, 1757 ± 367 cells/mm^2^ at 3 months, 1,740 ± 376 cells/mm^2^ at 6 months, and 1694 ± 404 cells/mm^2^ at 12 months postoperatively. The ECD loss rates were 33.7 ± 12.9% at 1 month, 35.0 ± 13.6% at 3 months, 36.0 ± 14.0% at 6 months, and 37.5 ± 14.5% at 12 months postoperatively. Immune rejection episodes were seen in 14 (1.66%) eyes. Re-bubbling procedures were performed in 328 (39.0%) eyes.

### Donor characteristics and comparison between organ culture and cold culture

As shown in Table 2, there were 644 (76.6%) organ culture donors and 197 (23.4%) cold culture donors. The mean donor age was 69.0 ± 10.9 years. The mean donor age in the organ culture group was 70.0 ± 0.4 years, which was significantly higher than that in the cold culture group (65.9 ± 0.8 years; P < 0.001; t-test). Regarding donor sex, 62.6% were men, and 37.4% were women. The mean storage time was 18.7 ± 0.2 days in the organ culture group, as opposed to 11.4 ± 0.4 days in the cold culture group. There was a significant difference between the organ and cold culture groups (P < 0.001; t-test).

**Table 2:** Comparison of the clinical course between the organ and cold culture groups.

Factors	Organ culture	Cold culture	P-value
Baseline ECD (cells/mm^2^) (mean ± SD)	2,734 ± 9 (n = 636)	2,663 ± 15 (n = 197)	< 0.001
ECD 1 month after surgery (cells/mm^2^) (mean ± SD)	1766 ± 20 (n = 287)	1872 ± 37 (n = 84)	0.012
ECD 3 months after surgery (cells/mm^2^) (mean ± SD)	1752 ± 23 (n = 246)	1773 ± 41 (n = 78)	0.661
ECD 6 months after surgery (cells/mm^2^) (mean ± SD)	1712 ± 21 (n = 303)	1833 ± 40 (n = 86)	0.001
ECD 12 months after surgery (cells/mm^2^) (mean ± SD)	1681 ± 21 (n = 357)	1741 ± 39 (n = 108)	0.179
ECD loss rates at 12 months (%)	38.3 ± 0.8 (n = 357)	34.7 ± 1.4 (n = 108)	0.022
Preoperative BCVA (LogMAR)	1.16 ± 0.03 (n = 527)	1.15 ± 0.06 (n = 156)	0.929
Postoperative BCVA (LogMAR) at 1 month	0.30 ± 0.01 (n = 562)	0.33 ± 0.02 (n = 172)	0.201
Postoperative BCVA (LogMAR) at 3 months	0.18 ± 0.01 (n = 396)	0.23 ± 0.02 (n = 116)	0.002
Postoperative BCVA (LogMAR) at 6 months	0.14 ± 0.01 (n = 391)	0.13 ± 0.01 (n = 107)	0.551
Postoperative BCVA (LogMAR) at 12 months	0.11 ± 0.01 (n = 394)	0.11 ± 0.01 (n = 114)	0.540
Preoperative CCT (μm)	661.1 ± 6.4 (n = 365)	666.4 ± 11.4 (n = 117)	0.865
Postoperative CCT at 1 month (μm)	554.9 ± 9.1 (n = 137)	594.2 ± 16.2 (n = 43)	0.036
Postoperative CCT at 3 months (μm)	534.7 ± 4.8 (n = 136)	525.0 ± 8.3 (n = 45)	0.315
Postoperative CCT at 6 months (μm)	538.4 ± 4.8 (n = 180)	516.7 ± 8.9 (n = 53)	0.034
Postoperative CCT at 12 months (μm)	535.3 ± 2.9 (n = 242)	532.8 ± 5.0 (n = 82)	0.667
Re-bubbling; yes (+) (%)	255 (40.0) (n = 637)	68 (34.5) (n = 197)	0.165
Immune rejection (+) (%)	7 (1.9) (n = 637)	7 (3.55) (n = 197)	0.019

*ECD* endothelial cell density, *SD* standard deviation, *BCVA* best-corrected visual acuity, *logMAR* logarithm of the minimal angle of resolution, *CCT* central corneal thickness.

The ECD in the organ culture was 2,734 ± 9 (baseline ECD; N = 636), 1766 ± 20 (1 month, N = 287), 1752 ± 23 (3 months, N = 246), 1712 ± 21 (6 months, N = 303), and 1681 ± 21 (12 months, N = 357); that in the cold culture was 2,663 ± 15 (baseline ECD, N = 197), 1872 ± 37 (1 month, N = 84), 1773 ± 41 (3 months, N = 78), 1833 ± 40 (6 months, N = 86), and 1741 ± 39 (12 months, N = 108). The ECD was significantly higher in the organ culture than in the cold culture at 1 month (P = 0.012, t-test) and 6 months postoperatively (P = 0.008, t-test). However, as the baseline ECD was significantly higher in the organ culture (P < 0.001, t-test), organ culture resulted in a significantly greater ECD loss (P < 0.001 at 1 month, P = 0.248 at 3 months, P = 0.003 at 6 months, and P = 0.022 at 12 months postoperatively; t-test).

**Figure 1:**
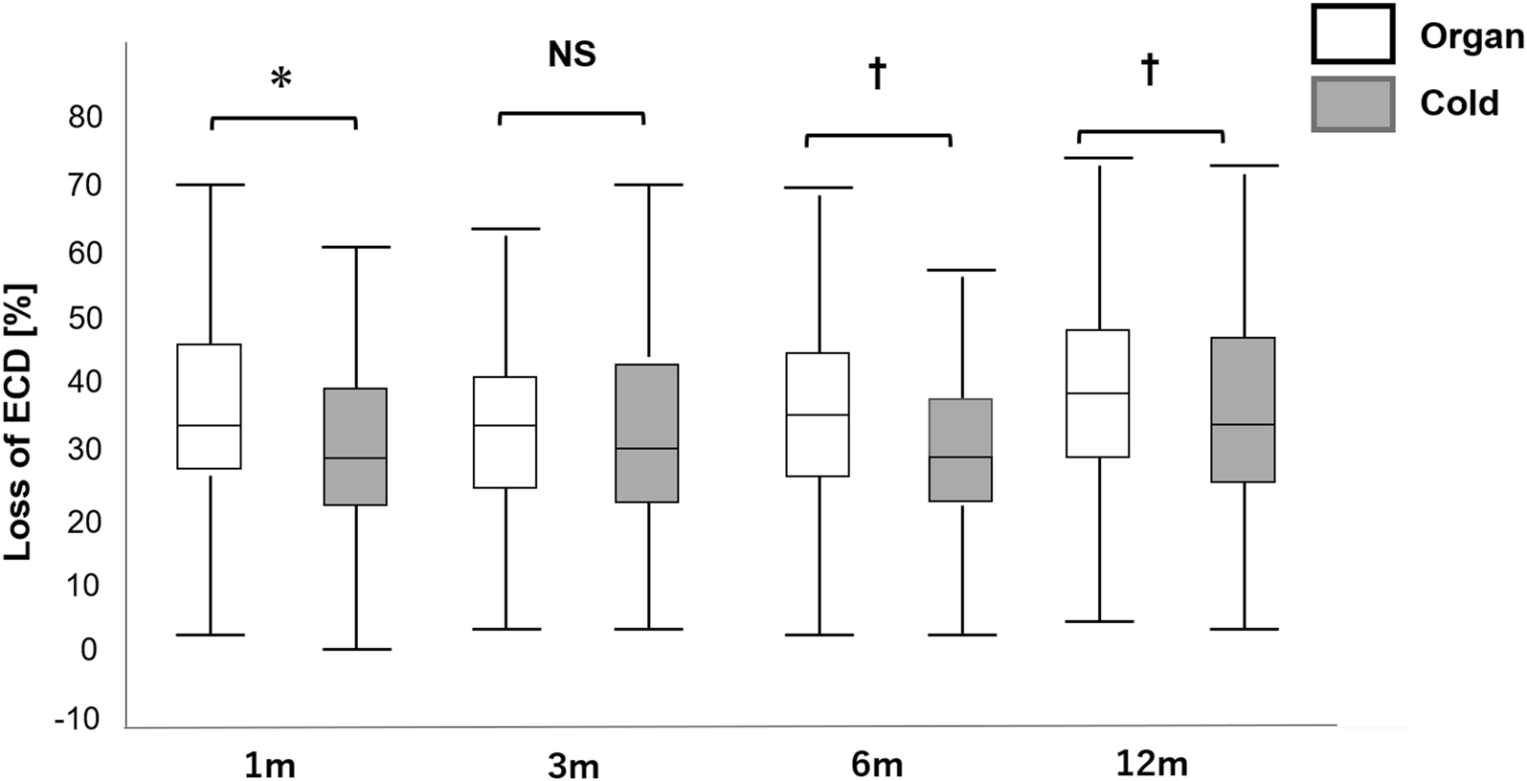
Comparison of ECD loss after DMEK between the organ and cold culture groups. The organ culture group had higher ECD loss rates than the cold culture group (*P < 0.001 at 1 month, P = 0.248 at 3 months, ^†^P < 0.05 [P = 0.003 at 6 months and P = 0.022 at 12 months] after surgery; t-test). Although there was a significant difference between the organ culture and cold culture groups, this difference is clinically irrelevant. The central line means median value, and the whiskers indicates the lowest and highest values. *ECD* endothelial cell density; *DMEK* Descemet membrane endothelial keratoplasty, *NS* not significant.

As shown in Fig. 1, the ECD loss rates in the organ culture were 33.5 ± 0.8% at 1 month (N = 287), 33.9 ± 0.9% at 3 months (N = 246), 35.6 ± 0.8% at 6 months (N = 302), and 36.8 ± 0.8% at 12 months (N = 357) postoperatively. Those in the cold culture were 26.6 ± 1.4% at 1 month (N = 84), 30.5 ± 1.5% at 3 months (N = 78), 29.2 ± 1.5% at 6 months (N = 86), and 31.9 ± 1.4% at 12 months (N = 108) postoperatively.

**Figure 2:**
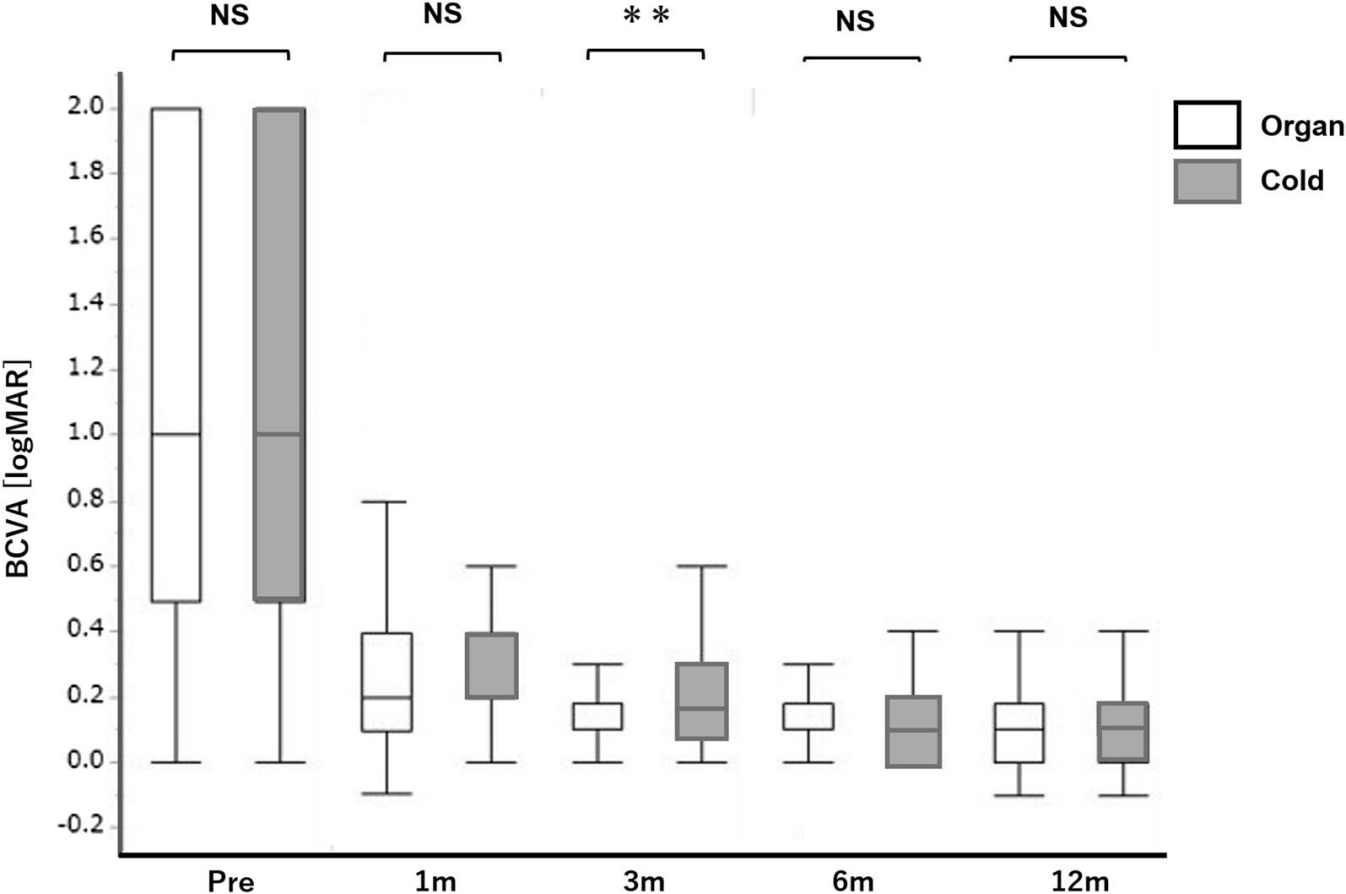
Comparison of the BCVA after DMEK between the organ and cold culture groups. There was a gradual improvement in the BCVA from baseline to 1, 3, 6, and 12 months after surgery. A significant difference was seen between the organ culture and cold culture groups only at 3 months after surgery (**P = 0.002; t-test). The central line means median value, and the whiskers indicates the lowest and highest values. *BCVA* best-corrected visual acuity, *DMEK* Descemet membrane endothelial keratoplasty.

**Figure 3:**
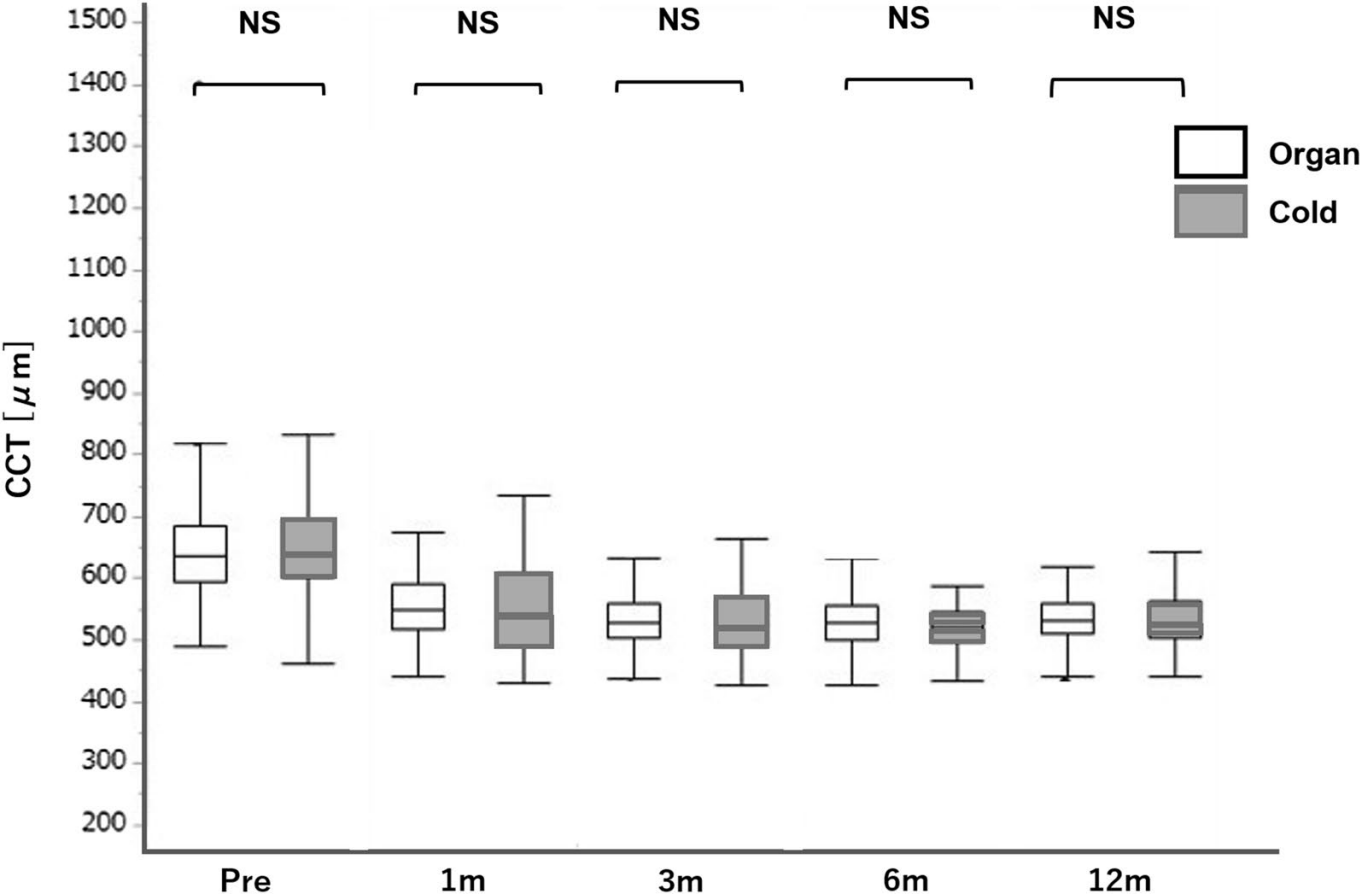
Comparison of the CCT after DMEK between the organ and cold culture groups. There was no significant difference between the organ culture and cold culture groups preoperatively and at 3 and 12 months postoperatively (P = 0.865, 0.315, and 0.667; t-test). The central line means median value, and the whiskers indicates the lowest and highest values. *CCT* central corneal thickness, *DMEK* Descemet membrane endothelial keratoplasty.

**Figure 4:**
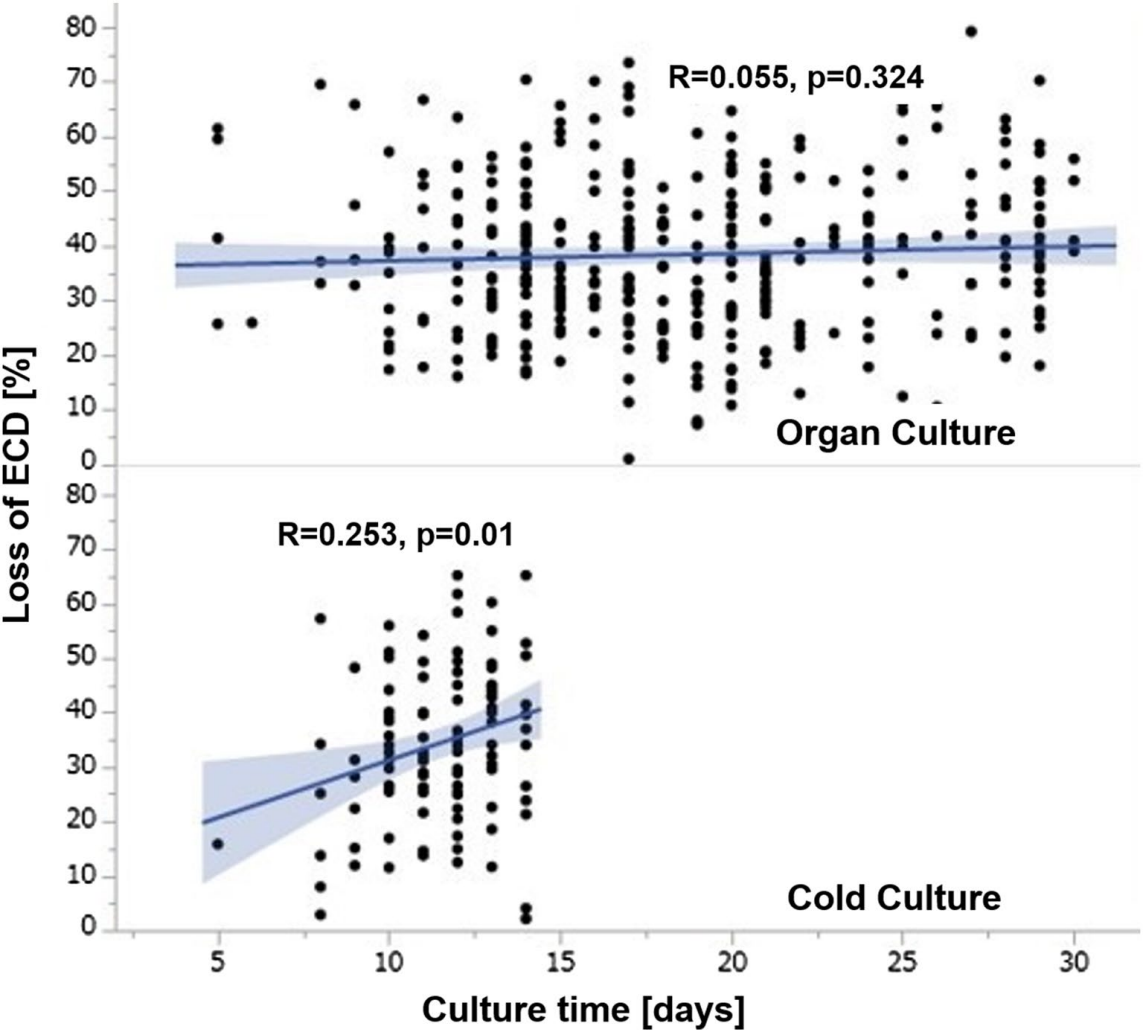
Correlation between the culture time and ECD decline in the organ and cold culture groups. There was no significant correlation between the culture time and ECD decline in the organ culture group (R = 0.0557, P = 0.324). The longer culture time had a negative impact on ECD decline in the cold culture group (R = 0.253, P = 0.01). *ECD* endothelial cell density.

The prognosis of the BCVA was similar in the two groups (Fig. 2), whereas a significant difference was noted only at 1 month postoperatively (P = 0.002, t-test). The transition of the CCT was also similar in the two groups (Fig. 3), whereas the CCT was significantly higher in the cold culture at 1 month postoperatively (P = 0.036). A longer culture time had a negative impact on ECD loss in the cold culture (Fig. 4).

There was no significant difference in re-bubbling (yes/no) between the two groups (P = 0.165, Pearson’s chi-squared test). The immune rejection rate was significantly higher in the cold culture (P = 0.019, Pearson’s chi-squared test). The percentage of the use of 20% SF6 gas was 46.3% in the organ culture and 55.8% in the cold culture (P < 0.001, Pearson’s chi-squared test).

### Correlation

As shown in Table 3 and Supplementary Tables S1 and S2 ECD loss at 1 year postoperatively was strongly associated with the occurrence of re-bubbling in all eyes (P < 0.001) and in those that received triple DMEK (P = 0.020) and DMEK alone (P < 0.001). Finally, re-bubbling was identified as an important risk factor after stepwise variable selection (P < 0.001).

**Table 3:** Factors associated with endothelial cell loss at 1 year after DMEK.

Evaluation factors	F ratio	P-value
Patient sex (female or male)	0.149	0.699
Patient age	3.849	0.050
Type of tamponade gas (air or 20% SF6 gas)	0.795	0.373
Type of surgery (triple DMEK or DMEK alone)	0.143	0.705
Re-bubbling (yes)	16.822	< 0.001
Immune rejection (yes)	1.106	0.294
Donor source (organ culture or cold culture)	2.908	0.889
Donor sex (female or male)	0.295	0.587
Donor age	3.009	0.084

*SF6*, sulphur hexafluoride, *DMEK* Descemet membrane endothelial keratoplasty, *triple DMEK* DMEK combined with cataract surgery and intra-ocular lens implantation, *DMEK alone* DMEK for pseudophakic eyes.

## Discussion

This retrospective study compared the clinical outcomes between organ culture and cold culture and evaluated the factors associated with ECD loss after DMEK. In general, reasonably good results were obtained at 1 year after DMEK in terms of ECD (1704 ± 399 cells/mm^2^) and ECD loss (37.0 ± 14.3%).

Some results from our comparison between organ culture and cold culture are not consistent with those from previous studies. Although the re-bubbling rates were higher in cold culture in previous studies^16–18^, there was no difference found in our study. Despite similar results for the BCVA and CCT, ECD loss seemed to be greater in the organ culture at 1, 6, and 12 months postoperatively. The difference in the values could be attributed to this divergence.

This study allows for the following conclusions to be drawn.

(1) Immune reactions are more common in cold cultures.

(2) Although organ culture yields higher ECD loss rates than does cold culture, the difference is clinically irrelevant.

(3) There is a strong correlation between the occurrence of re-bubbling and ECD loss at 1 year after DMEK.

Interestingly, the rate of immune rejection was significantly higher in the cold culture than in the organ culture (P = 0.009, Pearson’s chi-squared test). Some reports suggest the possibility that a longer storage time could decrease the immunogenicity of the donor and therefore decrease graft rejection^19,20^. A possible explanation for this could be that the outflow of bone marrow-derived antigen-presenting cells from the donor tissue during storage could suppress graft rejection. In fact, the storage time was longer in the organ culture than in the cold culture in our study. However, it is uncertain whether a longer storage time could reduce graft rejection in DMEK, in which only a monolayer of endothelium without donor bone marrow-derived antigen-presenting cells is transplanted.

A possible explanation for the greater ECD loss in the organ culture could be the longer storage time. In fact, the mean storage time in the organ culture (18.7 ± 0.2 days) was significantly longer than that in the cold culture (11.4 ± 0.4 days). However, this hypothesis was denied because there was no correlation found between the storage time and ECD loss in the organ culture, as shown in Fig. 4. Although there was no significant correlation between the culture time and ECD loss in the organ culture, the longer culture time had a negative impact on ECD loss in the cold culture. It is possible that the evaluation of the preoperative ECD is inaccurate. The lack of evaluation of ECD immediately before surgery could also be a limitation of this study because it has been reported that many dead cells can be detected after a long storage time^21–24^.

In our study, we performed a multivariable regression analysis to identify the risk factors for ECD loss. We found a strong relationship between re-bubbling and ECD loss. There was no significant difference in the occurrence of re-bubbling between the two groups (organ culture and cold culture). However, it remains uncertain whether re-bubbling procedures itself cause ECD loss or whether endothelial cells with low pump function (low viability or dead cells) cause graft detachment necessitating rebubbling.

Our multivariable regression analysis included some clinically important factors, such as the type of tamponade (air or 20% SF6 gas), the type of surgery (triple DMEK or DMEK alone), re-bubbling, immune rejection, and age and sex. Although the use of SF6 gas significantly reduced the rates of graft detachment and re-bubbling^15,21^, there was no significant difference in the ECD loss between SF6 and air. Although Busin et al*.* reported that triple DMEK could be a risk factor for re-bubbling^10^, the type of surgery was not found as a risk factor in our study.

The strengths of this study are the retrospective analysis within a prospective trial database for a single disease (FECD) in a large sample, determination of surgical outcomes by two experienced DMEK surgeons (both surgeons have experiences of > 4,000 DMEKs)^25^, and consideration of clinically important factors. In contrast, the limitations of this study include its retrospective nature, lack of precise donor information (death to harvest time), and missing values. Another limitation is the lack of precise evaluation of the graft/descemetorhexis size and actual times of re-bubbling. Feng et al*.* suggested that additional re-bubbling (more than twice) could have a negative impact on ECD loss^26^.

In conclusion, re-bubbling procedures or the innate graft viability that requires re-bubbling might have a negative impact on the final ECD outcome or ECD loss. Surgeons should reconsider the impact or the necessity of re-bubbling in such cases. Furthermore, both organ- and cold-donor culture media can be used safely in patients, as they have similar benefits.

## Methods

### Patient selection

Patients with FECD who underwent DMEK were selected from the Cologne DMEK database using Research Electronic Data Capture, which is a secure, website-based application designed to support data capture for research studies. The data of consecutive patients who underwent DMEK from 2011 to 2016 were obtained prospectively from medical records and reviewed via a retrospective analysis. The study was performed in accordance with the tenets of the Declaration of Helsinki; all study participants provided their informed consent; and the protocol was approved by the Ethics Commission of the Medical Faculty of the University of Cologne (number: 14-373). Although the FECD cases included those that received triple DMEK, DMEK alone, and phakic DMEK, those who underwent phakic DMEK were excluded because of their characteristics, such as younger age and a shallow anterior chamber (AC). Patients with pre-existing diseases/conditions that would influence the final visual acuity were also excluded (macular degeneration, wet-type AMD, amblyopia, history of trauma, uncontrolled glaucoma with advanced visual field loss, pre-existing AC lens, or history of glaucoma surgery, or vitrectomy). As for the patients with bilateral involvement, only the first eye was included. Cases of primary graft failure were also excluded from this study.

### Surgical technique and postoperative medication

As in our previous reports, standard DMEK procedures were performed by two experienced surgeons (CC and BB)^13,25^. Corneal donors were obtained from eye banks from Germany or the USA. In brief, the entire cornea was fixed with a suction block (Moria SA, Antomy, France) and stained with 0.06% trypan blue (Vision Blue, D.O.R.C. International, Zuidland, the Netherlands) for 5 to 10 s. The DM was peeled off and separated from the stroma. For proper orientation, asymmetric marking was used^27^ and punched in the proper diameter (8.0 to 10.0 mm). A DMEK graft was set in the intra-ocular lens (IOL) cartridge (AT Smart Cartridge, Carl Zeiss Meditec, Jena, Germany) and afterwards in the IOL injector (AT Shooter A2-2000, Zeiss Meditec)^28^. Under general anaesthesia, partial iridectomy was enlarged using Vannas capsulotomy scissors (Asico, Westmont, IL, USA), and descemetorhexis was performed under air within a diameter larger than the graft size using a Price hook^28^. The DMEK graft was placed into the AC, unfolded, and fixed with air or 20% SF6 gas (Alcon Laboratories, Fort Worth, TX, USA). In the triple DMEK cases, phacoemulsification and IOL implantation were performed before DMEK. Until air or gas disappeared, all the patients were instructed to assume the supine position under continuous monitoring of intra-ocular pressure. Pilocarpine 2% eye drops (Bausch & Lomb) were used three times a day. Postoperative medications included topical antibiotics (ofloxacin) (Floxal EDO; Mann Pharma, Berlin, Germany) administered four times daily for 2 weeks and prednisolone acetate 1% (Inflanefran Forte; Pharm-Allergan, Ettlingen, Germany) administered five times daily for the first postoperative month; steroids were then tapered once per month and administered for 24 months. (Briefly, patients received 5 drops per day in the first postoperative month, 4 drops per day in the second month postoperatively, 3 drops per day in the third month postoperatively, 2 drops per day in the fourth postoperative month and one drop per day from the fifth month postoperatively until two years after surgery.) Lubricant eye drops (Hylo Care; Ursapharm, Saarbrücken, Germany) were used for 5 days. The indication for re-bubbling was determined using an anterior segment OCT device (AS-OCT) within 14 days after surgery. In cases where the graft detachment reached the pupil area, the re-bubbling procedure was performed under the operating microscope in the operating room.

### Clinical data

The following donor information was included in the database: age (years), sex (female/male), ECD (cells/mm^2^), source of tissue, and storage time (days). The donor sources were divided into two groups: organ culture (stored in a Dulbecco modified Eagle’s medium containing streptomycin and penicillin [Biochrom, Berlin, Germany] as well as faetal calf serum [Linaris Bettingen am Main, Germany] at a warm temperature [37 ℃]) and cold culture (stored in Optisol-GS [Bauch & Lomb, Irvine, CA, USA] at a cold temperature [5 ℃]). In the organ culture group, the culture medium that consisted of Dulbecco modified Eagle’s medium with faetal calf serum was supplemented with dextran (10% dextran T500) medium before transplantation (average interval, 24–48 h). The storage time in both groups was defined from the timepoint when corneoscleral buttons were placed into the culture medium to the day of surgery.

After surgery, all patients attended follow-up visits as per standard protocols. In addition to the usual ophthalmic examination using slit-lamp microscopy and funduscopy of the retina, the following parameters were documented: BCVA (measured as decimal visual acuity and converted to logMAR units for statistical analysis), ECD [measured via specular microscopy of the corneal endothelium (EM-3000, Tomey, Nagoya, Japan)], CCT [measured via corneal tomography (Pentacam, Oculus, Wetzlar, Germany)], graft attachment [evaluated using the AS-OCT (Heidelberg Engineering GmbH, Germany)], and posterior segment values [evaluated using a macular SD-OCT (Spectralis HRAþOCT; Heidelberg Engineering GmbH)] before and 1, 3, 6, and 12 months after DMEK. A macular OCT was used for exclusion of the existence of macular degeneration or wet-type AMD. The preoperative (baseline) ECD was evaluated before storage.

### Evaluation factors and statistics

The clinical factors used for the analysis are numbered (1) to (9) below.

- The patient-related factors were as follows: (1) sex and (2) age.
- The procedure- and clinical course-related factors were as follows: (3) type of tamponade (air or 20% SF6 gas), (4) type of surgery (triple DMEK or DMEK alone), (5) re-bubbling (yes/no), and (6) immune rejection (yes).
- The donor-related factors were as follows: (7) sex, (8) age, and (9) source.

### Statistical analyses

Statistical analysis was performed using the JMP Pro software version 14.0.0 (SAS Institute, Cary, NC, USA). To compare the continuous variables in each group, we used the t-test; to compare the nominal variables, we applied Pearson’s chi-squared test. Pearson correlation analysis was used for the univariable analyses. Associations between the factors and ECD loss rates at 12 months were examined using multivariable regression analysis and second-order polynomial regression after stepwise variable selection (using the minimum Bayesian information criterion and increasing the number of variables). To exclude potential multicollinearity factors between the variables, we checked the variance inflation factor. The statistical significance level was set at P-values of < 0.05.

## Supplementary information

Supplementary Tables

## References

[1] Melles GR, Ong TS, Ververs B, van der Wees J (2006). Descemet membrane endothelial keratoplasty (DMEK). Cornea.

[2] Guerra FP, Anshu A, Price MO, Giebel AW, Price FW (2011). Descemet’s membrane endothelial keratoplasty: prospective study of 1-year visual outcomes, graft survival, and endothelial cell loss. Ophthalmology.

[3] Terry MA (2015). Standardized DMEK technique: reducing complications using Prestripped tissue, novel glass injector, and sulfur hexafluoride (SF6) gas. Cornea.

[4] Schlögl A, Tourtas T, Kruse FE, Weller JM (2016). Long-term clinical outcome after descemet membrane endothelial keratoplasty. Am. J. Ophthalmol..

[5] Flockerzi E (2018). Trends in corneal transplantation from 2001 to 2016 in Germany: a report of the DOG-section cornea and its Keratoplasty Registry. Am. J. Ophthalmol..

[6] Dapena I, Ham L, Netuková M, van der Wees J, Melles GR (2011). Incidence of early allograft rejection after descemet membrane endothelial keratoplasty. Cornea.

[7] Anshu A, Price MO, Price FW (2012). Risk of corneal transplant rejection significantly reduced with descemet’s membrane endothelial keratoplasty. Ophthalmology.

[8] Hos D (2017). Incidence and clinical course of immune reactions after descemet membrane endothelial keratoplasty: retrospective analysis of 1000 consecutive eyes. Ophthalmology.

[9] Hos D (2019). Immune reactions after modern lamellar (DALK, DSAEK, DMEK) versus conventional penetrating corneal transplantation. Prog. Retin. Eye Res..

[10] Busin M (2018). Clinical outcomes of preloaded descemet membrane endothelial keratoplasty grafts with endothelium tri-folded inwards. Am. J. Ophthalmol..

[11] Ang M (2016). Descemet membrane endothelial keratoplasty: preliminary results of a donor insertion pull-through technique using a donor mat device. Am. J. Ophthalmol..

[12] Ham L (2016). Midterm results of descemet membrane endothelial keratoplasty: 4 to 7 years clinical outcome. Am. J. Ophthalmol..

[13] Schrittenlocher S, Bachmann B, Cursiefen C (2019). Impact of donor tissue diameter on postoperative central endothelial cell density in Descemet membrane endothelial keratoplasty. Acta Ophthalmol..

[14] Terry MA, Shamie N, Straiko MD, Friend DJ, Davis-Boozer D (2011). Endothelial keratoplasty: the relationship between donor tissue storage time and donor endothelial survival. Ophthalmology.

[15] Schaub F (2017). One-year outcome after descemet membrane endothelial keratoplasty (DMEK) comparing sulfur hexafluoride (SF6) 20% versus 100% air for anterior chamber tamponade. Br. J. Ophthalmol..

[16] Salla S, Kruse FE, Walter P, Menzel-Severing J (2019). Supplementation of organ culture medium with dextran is not required in pre-stripped human donor tissue for DMEK surgery. Cell Tissue Bank.

[17] Abdin A (2018). Negative impact of dextran in organ culture media for pre-stripped tissue preservation on DMEK (Descemet membrane endothelial keratoplasty) outcome. Graefes Arch. Clin. Exp. Ophthalmol..

[18] Laaser K (2011). Donor tissue culture conditions and outcome after Descemet membrane endothelial keratoplasty. Am. J. Ophthalmol..

[19] Zhang X (2009). Depletion of passenger leukocytes from corneal grafts: an effective means of promoting transplant survival?. Invest. Ophthalmol. Vis. Sci..

[20] Kamiya K (2000). Preservation of donor cornea prevents corneal allograft rejection by inhibiting induction of alloimmunity. Exp. Eye Res..

[21] Güell JL, Morral M, Gris O, Elies D, Manero F (2015). Comparison of sulfur hexafluoride 20% versus air tamponade in Descemet membrane endothelial keratoplasty. Ophthalmology.

[22] Schrittenlocher S (2018). Evolution of consecutive Descemet membrane endothelial keratoplasty outcomes throughout a 5-year period performed by two experienced surgeons. Am. J. Ophthalmol..

[23] Kitazawa K (2017). The existence of dead cells in donor corneal endothelium preserved with storage media. Br. J. Ophthalmol..

[24] Rodríguez-Calvo de Mora M (2016). Association between graft storage time and donor age with endothelial cell density and graft adherence after descemet membrane endothelial keratoplasty. JAMA Ophthalmol..

[25] Stulting RD (2018). Cornea Preservation Time Study Group. Factors associated with graft rejection in the cornea preservation time study. Am. J. Ophthalmol..

[26] Feng MT, Price MO, Miller JM, Price FW (2014). Air reinjection and endothelial cell density in Descemet membrane endothelial keratoplasty: five-year follow-up. J. Cataract Refract. Surg..

[27] Bachmann BO, Laaser K, Cursiefen C, Kruse FE (2010). A method to confirm correct orientation of descemet membrane during descemet membrane endothelial keratoplasty. Am. J. Ophthalmol..

[28] Kruse FE (2011). A stepwise approach to donor preparation and insertion increases safety and outcome of descemet membrane endothelial keratoplasty. Cornea.

