# Supplementary material for: Risk factors for endothelial cell loss after Descemet membrane endothelial keratoplasty (DMEK)
Source: Sci Rep. 2020 Jul 6;10:11086. doi: 10.1038/s41598-020-68023-0 (PMC7338484; doi:10.1038/s41598-020-68023-0)
Supplement: Supplementary file 1 — Supplementary Tables [file 41598_2020_68023_MOESM1_ESM.pdf]

Risk factors for endothelial cell loss after Descemet membrane endothelial keratoplasty (DMEK)

Takahiko Hayashi,[1,2]* Silvia Schrittenlocher,[1] Sebastian Siebelmann,[1] Viet Nhat Hung Le,[1,3]
Mario Matthaei,[1] Jeremy Franklin,[4] Björn Bachmann,[1]** Claus Cursiefen[1,5]**

[1]Department of Ophthalmology, University of Cologne, Cologne, Germany
[2]Department of Ophthalmology, Yokohama Minami Kyosai Hospital, Kanagawa, Japan
[3]Department of Ophthalmology, Hue College of Medicine and Pharmacy, Hue University, Hue, Vietnam
[4]Institute of Medical Statistics and Computational Biology, University of Cologne, Cologne, Germany
[5]Centre for Molecular Medicine Cologne, CMMC, University of Cologne, Cologne, Germany

*Corresponding author:
Takahiko Hayashi, MD, PhD
Department of Ophthalmology, University of Cologne
Kerpener Str. 62, Cologne 50937, Germany
Tel: (+49) 221-4313
Fax: (+49) 221-3186
Email: takamed@gmail.com

**Co-senior authors

Supplementary Table 1. The Factors Associated with Endothelial Cell Loss at one year after Triple DMEK.

**Evaluating factors**	**F ratio**	***P*–Value**
Patient gender (Female or Male)	0.048	0.828
Patient age	3.454	0.064
Type of tamponade gas (Air or 20 % SF6 Gas)	1.778	0.183
Rebubbling (Yes)	5.488	0.020
Immune Rejection (Yes)	1.534	0.217
Donor source (Organ culture or Cold culture)	1.056	0.305
Donor gender (Female or Male)	0.916	0.339
Donor age	1.423	0.234

Rebubbling has a significant correlation in multiple analysis (p=0.020).

Triple DMEK, DMEK combined with cataract surgery, intraocular lens (IOL) implantation.
SF6 Gas, sulfur hexafluoride 6;
DMEK, Descemet Membrane Endothelial Keratoplasty;

Supplementary Table 2. The Factors Associated with Endothelial Cell Loss at one year after DMEK alone.

**Evaluating factors**	**F ratio**	***P*–Value**
Patient gender (Female or Male)	0.043	0.835
Patient age	3.152	0.078
Type of tamponade gas (Air or 20 % SF6 Gas)	0.014	0.906
Rebubbling (Yes)	13.16	< 0.001
Immune Rejection (Yes)	0.743	0.390
Donor source (Organ culture or Cold culture)	3.287	0.072
Donor gender (Female or Male)	0.313	0.577
Donor age	2.324	0.129

Rebubbling has a significant correlation in multiple analysis ($p < 0.001$).

DMEK, Descemet Membrane Endothelial Keratoplasty;
DMEK alone, DMEK for pseudophakic eyes;
SF6 Gas, sulfur hexafluoride 6;
